# Systematic review on diabetes mellitus and dental implants: an update

**DOI:** 10.1186/s40729-021-00399-8

**Published:** 2022-01-03

**Authors:** Juliane Wagner, Johannes H. Spille, Jörg Wiltfang, Hendrik Naujokat

**Affiliations:** Department of Oral and Maxillofacial Surgery, University-Hospital Schleswig-Holstein, Campus Kiel, 24105 Kiel, Germany

**Keywords:** Dental implants, Implant survival, Diabetes mellitus, Prediabetes, Glycemic control, Peri-implantitis, Systemic inflammation, Systemic disease, Risk factor

## Abstract

**Purpose:**

Dental implant surgery was developed to be the most suitable and comfortable instrument for dental and oral rehabilitation in the past decades, but with increasing numbers of inserted implants, complications are becoming more common. Diabetes mellitus as well as prediabetic conditions represent a common and increasing health problem (International Diabetes Federation in IDF Diabetes Atlas, International Diabetes Federation, Brussels, 2019) with extensive harmful effects on the entire organism [(Abiko and Selimovic in Bosnian J Basic Med Sci 10:186–191, 2010), (Khader et al., in J Diabetes Complicat 20:59–68, 2006, 10.1016/j.jdiacomp.2005.05.006)]. Hence, this study aimed to give an update on current literature on effects of prediabetes and diabetes mellitus on dental implant success.

**Methods:**

A systematic literature research based on the PRISMA statement was conducted to answer the PICO question “Do diabetic patients with dental implants have a higher complication rate in comparison to healthy controls?”. We included 40 clinical studies and 16 publications of aggregated literature in this systematic review.

**Results:**

We conclude that patients with poorly controlled diabetes mellitus suffer more often from peri-implantitis, especially in the post-implantation time. Moreover, these patients show higher implant loss rates than healthy individuals in long term. Whereas, under controlled conditions success rates are similar. Perioperative anti-infective therapy, such as the supportive administration of antibiotics and chlorhexidine, is the standard nowadays as it seems to improve implant success. Only few studies regarding dental implants in patients with prediabetic conditions are available, indicating a possible negative effect on developing peri-implant diseases but no influence on implant survival.

**Conclusion:**

Dental implant procedures represent a safe way of oral rehabilitation in patients with prediabetes or diabetes mellitus, as long as appropriate precautions can be adhered to. Accordingly, under controlled conditions there is still no contraindication for dental implant surgery in patients with diabetes mellitus or prediabetic conditions.

## Background

Nowadays, oral rehabilitation is increasingly achieved through the insertion of dental implants. This takes into account the patient’s and practitioner’s growing desire for aesthetically and chewing-functionally demanding as well as minimally invasive solutions with a high durability. Nevertheless, with increasing numbers of inserted implants, complications are becoming more common. A sufficient osseointegration of the previously placed implants is inevitable for early implant survival. During the osseointegration, however, bone remodeling plays an increasingly crucial role for implant success.

Diagnostic criteria for diabetes mellitus are a fasting plasma glucose in venous plasma with a concentration of ≥ 126 mg/dL, a HbA1c ≥ 6.5%, a 2-h postload plasma glucose measurement of ≥ 200 mg/dL or a random plasma glucose ≥ 200 mg/dL in the presence of symptoms of hyperglycaemia, such as polydipsie or polyurie [1]. Prediabetic conditions are defined as an intermediate hyperglycaemia, that do no attain diabetes thresholds [2]. However, both are very common metabolic disorders, that cause hyperglycemia leading to micro- and macroangiopathies [3]. They are known to be associated with periodontitis [4], delayed wound healing [5] and an impairments of bone metabolism [6].

Diabetes mellitus as well as prediabetic conditions represent a common and increasing health problem with extensive harmful effects on the entire organism. Although diabetes mellitus has been regarded as a relative risk factor for dental rehabilitation with implants, dental implant surgery was developed to be the most suitable and comfortable instrument for dental and oral rehabilitation in the past decades.

Hence, this systematic review aimed to give an update on current literature on effects of pre-diabetes and diabetes mellitus on dental implant success, especially on postoperative complications, peri-implantitis and implant failure rates.

## Materials and methods

The substructure of the systematic review is based on the PRISMA 2020 statement/checklist (Table 1) [7]. The focused question was built according to the PICO (population, intervention, comparison, outcome) scheme. It answers the questions “Who are the patients?—diabetic patients” for “P” or population, “What are they exposed to?—dental implants” for “I” or intervention, “What do we compare them to?—healthy controls” for “C” or comparison and for “O” or outcome “What is the outcome?—the complication rate”. Accordingly, the focused question is: “Do diabetic patients with dental implants have a higher complication rate in comparison to healthy controls?”. A registration has not been performed and no review protocol has been prepared.

**Table 1:** PRISMA checklist

Section and topic	Item #	Checklist item	Location where item is reported
*Title*			
Title	1	Identify the report as a systematic review	Headline
*Abstract*			
Abstract	2	See the PRISMA 2020 for Abstracts checklist	–
*Introduction*			
Rationale	3	Describe the rationale for the review in the context of existing knowledge	Last sentence of introduction
Objectives	4	Provide an explicit statement of the objective(s) or question(s) the review addresses	Last sentence of introduction
*Methods*			
Eligibility criteria	5	Specify the inclusion and exclusion criteria for the review and how studies were grouped for the syntheses	M&M, Study inclusion and exclusion criteria
Information sources	6	Specify all databases, registers, websites, organisations, reference lists and other sources searched or consulted to identify studies. Specify the date when each source was last searched or consulted	M&M, search strategies
Search strategy	7	Present the full search strategies for all databases, registers and websites, including any filters and limits used	M&M, search strategies
Selection process	8	Specify the methods used to decide whether a study met the inclusion criteria of the review, including how many reviewers screened each record and each report retrieved, whether they worked independently, and if applicable, details of automation tools used in the process	M&M, search strategies, first sentence
Data collection process	9	Specify the methods used to collect data from reports, including how many reviewers collected data from each report, whether they worked independently, any processes for obtaining or confirming data from study investigators, and if applicable, details of automation tools used in the process	M&M, search strategies, first sentence
Data items	10a	List and define all outcomes for which data were sought. Specify whether all results that were compatible with each outcome domain in each study were sought (e.g., for all measures, time points, analyses), and if not, the methods used to decide which results to collect	M&M, search strategies, second sentence
	10b	List and define all other variables for which data were sought (e.g., participant and intervention characteristics, funding sources). Describe any assumptions made about any missing or unclear information	M&M, search strategies, second sentence
Study risk of bias assessment	11	Specify the methods used to assess risk of bias in the included studies, including details of the tool(s) used, how many reviewers assessed each study and whether they worked independently, and if applicable, details of automation tools used in the process	M&M, Quality and risk of bias assessment of selected studies; Tables 2/3
Effect measures	12	Specify for each outcome the effect measure(s) (e.g., risk ratio, mean difference) used in the synthesis or presentation of results	No effect measures were used due to heterogenous study designs
Synthesis methods	13a	Describe the processes used to decide which studies were eligible for each synthesis (e.g., tabulating the study intervention characteristics and comparing against the planned groups for each synthesis (item #5))	M&M, study selection, Sentence 6
	13b	Describe any methods required to prepare the data for presentation or synthesis, such as handling of missing summary statistics, or data conversions	M&M, Quality and risk of bias assessment of selected studies
	13c	Describe any methods used to tabulate or visually display results of individual studies and syntheses	M&M, Quality and risk of bias assessment of selected studies, last paragraph
	13d	Describe any methods used to synthesize results and provide a rationale for the choice(s). If meta-analysis was performed, describe the model(s), method(s) to identify the presence and extent of statistical heterogeneity, and software package(s) used	M&M, Quality and risk of bias assessment of selected studies, last paragraph
	13e	Describe any methods used to explore possible causes of heterogeneity among study results (e.g., subgroup analysis, meta-regression)	M&M, Quality and risk of bias assessment of selected studies, last paragraph
	13f	Describe any sensitivity analyses conducted to assess robustness of the synthesized results	No sensitivity analysis has been performed
Reporting bias assessment	14	Describe any methods used to assess risk of bias due to missing results in a synthesis (arising from reporting biases)	M&M, Quality and risk of bias assessment of selected studies, Risk of bias tools
Certainty assessment	15	Describe any methods used to assess certainty (or confidence) in the body of evidence for an outcome	M&M, Quality and risk of bias assessment of selected studies, Clinical studies, penultimate paragraph; Table 3
*Results*			
Study selection	16a	Describe the results of the search and selection process, from the number of records identified in the search to the number of studies included in the review, ideally using a flow diagram	Figure 1
	16b	Cite studies that might appear to meet the inclusion criteria, but which were excluded, and explain why they were excluded	Figure 1, Results, Study selection, 3rd section
Study characteristics	17	Cite each included study and present its characteristics	Table 6
Risk of bias in studies	18	Present assessments of risk of bias for each included study	Tables 2/3/5
Results of individual studies	19	For all outcomes, present, for each study: (a) summary statistics for each group (where appropriate) and (b) an effect estimate and its precision (e.g., confidence/credible interval), ideally using structured tables or plots	Table 6
Results of syntheses	20a	For each synthesis, briefly summarise the characteristics and risk of bias among contributing studies	Tables 2/3/5
	20b	Present results of all statistical syntheses conducted. If meta-analysis was done, present for each the summary estimate and its precision (e.g., confidence/credible interval) and measures of statistical heterogeneity. If comparing groups, describe the direction of the effect	No statistical analysis has been performed
	20c	Present results of all investigations of possible causes of heterogeneity among study results	Tables 3/5
	20d	Present results of all sensitivity analyses conducted to assess the robustness of the synthesized results	No sensitivity analysis has been performed
Reporting biases	21	Present assessments of risk of bias due to missing results (arising from reporting biases) for each synthesis assessed	M&M, Quality and risk of bias assessment of selected studies, Clinical studies
Certainty of evidence	22	Present assessments of certainty (or confidence) in the body of evidence for each outcome assessed	Table 3
*Discussion*			
Discussion	23a	Provide a general interpretation of the results in the context of other evidence	Conclusion section
	23b	Discuss any limitations of the evidence included in the review	First part of the conclusion
	23c	Discuss any limitations of the review processes used	First part of the conclusion
	23d	Discuss implications of the results for practice, policy, and future research	Conclusion, last part
*Other information*			
Registration and protocol	24a	Provide registration information for the review, including register name and registration number, or state that the review was not registered	M&M, first part
	24b	Indicate where the review protocol can be accessed, or state that a protocol was not prepared	M&M, first part
	24c	Describe and explain any amendments to information provided at registration or in the protocol	–
Support	25	Describe sources of financial or non-financial support for the review, and the role of the funders or sponsors in the review	No fundings/Funding section
Competing interests	26	Declare any competing interests of review authors	No conflicts of interest/Competing interests section
Availability of data, code and other materials	27	Report which of the following are publicly available and where they can be found: template data collection forms; data extracted from included studies; data used for all analyses; analytic code; any other materials used in the review	M&M, search strategies

From: Page MJ, McKenzie JE, Bossuyt PM, Boutron I, Hoffmann TC, Mulrow CD, et al. The PRISMA 2020 statement: an updated guideline for reporting systematic reviews. BMJ 2021;372:n71. 10.1136/bmj.n71; For more information, visit: http://www.prisma-statement.org/

**Table 2:** Risk of bias assessment for clinical studies

Risk of bias assessment	Cochrane risk of bias tool I	New Castle–Ottawa Scale	Based on Moga et al. (2012)	Number of studies
High risk of bias	< 3	< 4	< 2	4
Moderate risk of bias	3–5	4–6	2–3	9
Low risk of bias	6–8	7–9	4	26

**Table 3:** GRADE quality rating for clinical studies

Study (author/year)	(a) Risk of bias	(b) Indirectness	(c) Heterogenity	(d) Lack of precision	(e) Publication bias	GRADE quality rating
Eskow et al. (2017) [10]	Low	No	No	No data given	No	+
Ormianer et al. (2018) [11]	Moderate	No	No	No	No	+
Castellanos-Cosano et al. (2019) [12]	Low	No	No	No data given	No	++
Alrabiah et al. (2019) [13]	Low	No	No	No data given	No	++
Sghaireen et al. (2020) [14]	Low	No	No	No	No	+++
Papantonopoulos et al. (2017) [15]	Low	No	No	No data given	No	++
Atarchi et al. (2020) [16]	Moderate	No	No	No	No	+
Alasqah et al. (2018) [17]	Low	No	No	No data given	No	++
Singh et al. (2020) [18]	High	No	No	No data given	No	+
Al Zahrani et al. (2019) [19]	Low	No	No	No data given	No	++
Erdogan et al. (2015) [20]	Low	No	No	No data given	No	++
Oztel et al. (2017) [21]	Moderate	No	Yes	No	Possible	+
Gomez-Moreno et al. (2015) [22]	Low	No	Nein	No data given	No	++
Dogan et al. (2015) [23]	Low	No	Nein	No data given	No	++
Okamoto et al. (2018) [24]	Low	No	No	No	No	+++
Al Amri et al. (2015) [25]	Low	No	No	No	No	+++
de Araujo Nobre et al. (2016)	Low	No	No	No	No	+
Al Amri et al. (2017) [26]	Low	No	No	No data given	No	++
Al Amri et al. (2017) [27]	Low	No	No	No data given	No	++
Soh et al. (2020) [28]	Moderate	No	No	No data given	No	+
Mohanty et al. (2018) [29]	High	No	No	No data given	No	+
Aguilar-Salvatierra et al. (2016) [30]	Low	No	No	No data given	No	++
Rekawek et al. (2021) [31]	Low	No	No	No	No	+++
Jagadeesh et al. (2020) [32]	High	No	No	No data given	Possible	+
Kandasamy et al. (2018) [33]	Moderate	No	No	No data given	Possible	+
Pedro et al. (2017) [34]	Moderate	No	No	No data given	No	+
Yadav et al. (2018) [35]	Low	No	No	No data given	No	++++
Khan et al. (2016) [36]	High	No	No	No data given	No	+
French et al. (2021) [37]	Moderate	No	No	No	No	+
Alberti et al. (2020) [38]	Low	No	No	No	No	+++
Krebs et al. (2019) [39]	Low	No	No	No	No	+
Dalago et al. (2017) [40]	low	no	no	No	No	+
De Araújo Nobre et al. (2017) [41]	Moderate	No	No	No	No	+
Mayta-Tovalino et al. (2019) [42]	Moderate	No	No	No	No	+
Kissa et al. (2020) [43]	Low	No	No	No	No	+
Krennmair et al. (2018) [44]	Low	No	No	No	No	+
Al-Sowygh et al. (2018) [45]	Low	No	No	No data given	No	++
Corbella et al. (2020) [46]	Low	No	No	No	No	+
Al Amri et al. (2017) [47]	Low	No	No	No data given	No	++
Weinstein et al. (2020) [48]	Low	No	No	No	No	+

**Fig. 1:**
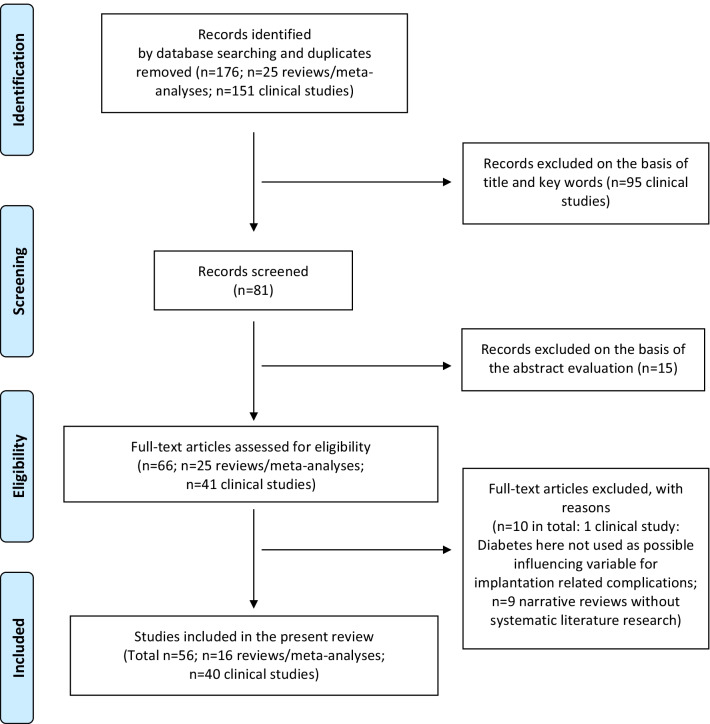
Flowchart of identified, excluded and included literature

### Search strategies

The systematic literature search and data extraction were performed by two independent scientists (JWa and HN). The following databases were incorporated in the systematic search for relevant literature: PubMed, AWMF Online and Cochrane Library. The following search terms were used: *dental implant AND diabetes, transgingival implant AND diabetes, maxillary augmentation AND diabetes, mandibular augmentation AND diabetes, periimplantitis AND diabetes, Zahnimplantate AND Diabetes, Kieferkammaufbau AND Diabetes*, *dental implant AND prediabetes, transgingival implant AND prediabetes, maxillary augmentation AND prediabetes, mandibular augmentation AND prediabetes, periimplantitis AND prediabetes, Zahnimplantate AND Prädiabetes, Kieferkammaufbau AND Prädiabetes*. Electronic search was complemented by an iterative hand-search in the reference lists of the already identified articles. The search for aggregated literature was carried out analogously to the search for the clinical literature described above. In addition to the search criteria, the filters meta-analysis, review and systematic review were used and the search was carried out using the above search criteria with the addition meta-analysis or AND meta-analysis or AND Review or AND Systematic Review. Electronic search was complemented by an iterative hand-search in the reference lists of the already identified articles. The starting point of the search was May 7th 2015, taking the time period of our prior literature research and publication into consideration [8]. The end point of the search was April 23rd 2021. Publications before and after these dates have not been considered. A total of 151 of clinical literature studies and 25 studies of aggregated literature were identified after removing duplicates. A total of 25 duplicates were excluded at the title level (Fig. 1). Endnote X9 was used for the electronic management of the literature.

### Study inclusion and exclusion criteria

Studies at abstract level were included according to the following criteria:

1. English or German language.
2. Retrospective and prospective clinical interventional and observation studies, cross-sectional studies, cohort studies, case series.

During the abstract review, hits were excluded according to the following criteria:

1. In vitro studies.
2. Animal studies.
3. Case reports with fewer than 10 patients.

During the assessment the full text of the aggregated literature was excluded according to the following criteria:

Diabetes mellitus/prediabetes not an influencing factor for implant-related parameters.

During the assessment the full text of the aggregated literature was excluded according to the following criteria:

1. Narrative reviews.
2. Reviews without systematic literature research.

### Quality and risk of bias assessment of selected studies

#### Clinical studies

The assessment of the internal validity of the primary literature was carried out in the only randomized controlled trial (RCT) presented here, using the Cochrane Risk of Bias Tool I. Here, the assessment was based on six higher level types of bias (a total of eight sub-items).

1. Selection bias: has the randomization been carried out adequately? Has the allocation been made in a blinded manner (allocation concealment)?
2. Performance bias: has the patient and staff been blinded?
3. Detection bias: was the evaluation blinding?
4. Attrition bias: has the adequate handling of missing result data been adequately described?
5. Reporting bias: were planned endpoints really reported?
6. Other bias: is there no other source of bias?

The selection bias, reporting bias and other bias were assessed for the entire study. The performance bias, detection bias and attrition bias were determined based on endpoints. The only RCT included showed an overall low risk of bias, as six out of eight sub-items could be answered with yes.

The assessment of the internal validity of the 19 cohort studies was based on the New Castle–Ottawa Scale (NOS). Three overarching areas were addressed with a total of nine questions:

- (1) Selection: were the selected cases adequately described (patient characteristics including risk factors, did the consecutive inclusion take place?
  - Are the cases representative of the average population?
  - Can you describe the collective in an understandable way?
  - Is the intervention (everything that has an impact on the outcome) adequately described?
  - Has the intervention (everything that has an influence on the outcome) been adequately surveyed?
- (2) Comparability: are controls and cases comparable? Are influencing factors checked? Are the results adjusted?
- (3) Outcome.
  - Is the outcome adequately described?
  - Is the outcome adequately recorded?
  - Has the follow-up been chosen long enough?
  - Is the number of patients (in follow-up) high enough?

For the assessment of the risk of bias in the cohort studies, a maximum of nine stars are awarded if the questions are answered positively. A maximum of four stars can be achieved for the area of selection bias, a maximum of two stars for comparability and a maximum of three stars for outcome.

The internal validity of the present 18 case series was based on Moga et al. 2012 [9].

The following four questions were addressed and answered with yes, partially, unclear or no:

1. Were the cases adequately described?
2. Has the intervention been adequately described and has the relevant data been adequately collected?
3. Have the outcomes been described adequately and was the relevant data collected adequately?
4. Has the follow-up period been chosen long enough?

A maximum of four points could be achieved in this way. The final assessment of the risk of bias was then carried out as shown in the following table (Table 2).

The assessment of the risk of bias was then included in the assessment of the evidence (“Body of Evidence”) according to GRADE (“Grades of Recommendation, Assessment, Development and Evaluation”). In addition, the indirectness (missing mapping of the PICO elements), the heterogeneity of the results and inconsistencies, a lack of precision as well as the suspicion or evidence of publication bias were also included in the evaluation of the quality according to GRADE. A downgrading of one level (“serious”) or two levels (“very serious”) per aspect is possible. With a devaluation of two levels, the maximum achievable evidence is moderate. Cohort studies were upgraded with a low risk of bias and positive evaluation of all other criteria included in GRADE. High quality (++++) rating was achieved by the RCT of Yadav et al. 2018, five studies achieved a moderate quality (+++) as they were upgraded cohort studies, low quality (++) was assumed for 13 cohort studies. In total 20 case studies as well as downgraded cohort studies only a achieved a very low (+) GRADE quality rate (Table 3).

No studies were excluded due to a lack of quality, but all data were included in the evaluation.

Moreover, the external validity of the available clinical literature was determined, as the question, whether the results can be transferred to the German supply situation, was answered. Attention was paid to the collective of patients, the treatment plan used and the setting (Table 4).

**Table 4:** External validity for clinical studies

Study (author/year)	Results transferable to the German supply situation?		
	Patients	Treatment	Setting
Eskow et al. (2017) [10]	Yes	Yes	Yes
Ormianer et al. (2018) [11]	Yes	Yes	Yes
Castellanos-Cosano et al. (2019) [12]	Yes	Yes	Yes
Alrabiah et al. (2019) [13]	Yes	Yes	Yes
Sghaireen et al. (2020) [14]	Yes	Yes	Yes
Papantonopoulos et al. (2017) [15]	Yes	Yes	Yes
Atarchi et al. (2020) [16]	Yes	Yes	Yes
Alasqah et al. (2018) [17]	Yes	Yes	Yes
Singh et al. (2020) [18]	Yes	Yes	Yes
Al Zahrani et al. (2019) [19]	Yes	Yes	Yes
Erdogan et al. (2015) [20]	Yes	Yes	Yes
Oztel et al. (2017) [21]	Yes	Yes	Yes
Gomez-Moreno et al. (2015) [22]	Yes	Yes	Yes
Dogan et al. (2015) [23]	Yes	Yes	Yes
Okamoto et al. (2018) [24]	Uncertain	Yes	Uncertain, obviously university for women
Al Amri et al. (2015) [25]	Male subjects only	Yes	Yes
de Araujo Nobre et al. (2016)	Yes	Yes	Yes
Al Amri et al. (2017) [26]	Yes	Yes	Yes
Al Amri et al. (2017) [27]	Male subjects only	Yes	Yes
Soh et al. (2020) [28]	Unclear	Unclear	Unclear
Mohanty et al. (2018) [29]	Unclear	Unclear	Unclear
Aguilar-Salvatierra et al. (2016) [30]	Yes	Yes	Yes
Rekawek et al. (2021) [31]	Yes	Yes	Yes
Jagadeesh et al. (2020) [32]	Yes	n.d.	Yes
Kandasamy et al. (2018) [33]	Yes	n.d.	Yes
Pedro et al. (2017) [34]	Yes	n.d.	n.d.
Yadav et al. (2018) [35]	Yes	Yes	Yes
Khan et al. (2016) [36]	Yes	n.d.	n.d.
French et al. (2021) [37]	Yes	Yes	Yes
Alberti et al. (2020) [38]	Yes	Yes	Yes
Krebs et al. (2019) [39]	Yes	Yes	Yes
Dalago et al. (2017) [40]	Yes	Yes	Yes
De Araújo Nobre et al. (2017) [41]	Yes	Yes	Yes
Mayta-Tovalino et al. (2019) [42]	Yes	Yes	Yes
Kissa et al. (2020) [43]	Yes	Yes	Yes
Krennmair et al. (2018) [44]	Yes	Yes	Yes
Al-Sowygh et al. (2018) [45]	Yes	Yes	Yes
Corbella et al. (2020) [46]	Yes	Yes	Yes
Al Amri et al. (2017) [47]	Male subjects only	Yes	Yes
Weinstein et al. (2020) [48]	Yes	Yes	Yes

*n.d.* no data provided

#### Aggregated literature

The assessment of the aggregated literature was based on the AMSTAR (Assessment of Multiple SysTemAtic Reviews)-2 criteria, including eleven questions that can be answered with yes, no, uncertain or not applicable. If a question is answered with yes, one point will be awarded. A maximum of eleven points could be achieved per study. The following 11 questions were used to assess the quality:

1. A priori planning/definition: Do you refer to a protocol or previously defined research goals?
2. Was the study selection and data extraction carried out by two independent persons?
3. Has the comprehensive and systematic literature search been carried out?
4. Have unpublished data/grey literature been considered?
5. Are the references for included and excluded studies given in the review article? Are the references listed and accessible electronically?
6. Were the study characteristics (patient characteristics, intervention (s) and endpoints) of the included studies given in tabular form or in detail in text form?
7. Was the risk of bias of the included primary studies assessed using established methods?
8. Was the risk of bias of the included studies considered for the result interpretation of the review article? (No yes, if previous question was not answered with yes)
9. Were the study results statistically adequately evaluated? Have pooled results been determined? Have heterogeneity tests been carried out?
10. Have publication bias/dissemination bias been addressed? Have at least ten primary studies been included?
11. Have any conflicts of interest been addressed?

The quality was then assessed using a scale based on the following points: 0–3 points: low quality; 4–7 points: moderate quality; 8–11 points: high quality [49]. Based on this rating, the quality of 15 studies was rated as high. The evaluation of two studies as moderate and no studies with a low quality. No studies had to be excluded due to a low quality (Table 5).

**Table 5:** AMSTAR-quality rating for aggregated literature due to AMSTAR-2 criteria

Study (first author/year)	(1) A priori planning/definition?	(2) Was the study selection and data extraction carried out by two independent persons?	(3) Systematic literature search	(4) Has grey literature been taken into account?	(5) References given and electronicall available?	(6) Study characteristics given?	(7) Risk of bias assessment?	(8) Was the risk of bias taken into account for interpretation in the review article?	(9) Adequate statistics? Pooled results? Heterogenity tests?	(10) Have publication bias/dissemination bias been addressed? Have at least ten primary studies been included?	(11) Have conflicts of interest been addressed?	AMSTAR-Rating	AMSTAR-Quality (8–11 = high, 4–7 = medium; 0–3 = low)
Naujokat et al. (2016) [8]	y	y	y	n	y	y	y	y	n	y	y	9	High
Jiang et al. (2021) [50]	y	u	y	n	y	y	y	y	y	y	y	9	High
Moraschini et al. (2016) [51]	y	y	y	y	y	y	y	y	y	y	y	11	High
Schimmel et al. (2018) [52]	y	y	y	u	y	y	y	y	y	y	y	10	High
Singh et al. (2019) [53]	y	u	y	n	y	y	n	n	n	y	y	6	Medium
Ting et al. (2018) [54]	y	y	y	u	y	y	y	y	u	y	y	9	High
Souto-Maior et al. (2019) [55]	y	y	y	u	y	y	y	y	n	n	y	8	High
De Oliveira-Neto et al. (2019) [56]	y	u	y	y	y	y	y	y	y	n	y	9	High
Shi et al. (2016) [57]	y	y	y	n	y	y	y	u	y	n	y	8	High
Shang et al. (2021) [58]	y	y	y	n	y	y	y	y	y	n	y	9	High
Lagunov et al. (2019) [59]	y	y	y	n	y	y	y	y	y	n	n	8	High
Dreyer et al. (2018) [60]	y	y	y	n	y	n	y	y	y	n**	y	8	High
Monje et al. (2017) [61]	y	y	y	y	y	y	y	u	y	y	y	10	High
Turri et al. (2016) [62]	y	u*	y	n	y	y	y	u	u	n	y	6	Medium
Meza Mauricio et al. (2019) [63]	y	y	y	n	y	y	y	n	n	y	y	8	High
Guobis et al. (2016) [64]	y	y	y	n	y	y	y	y	n	n**	n	7	High

*y* yes, *n* no, *u* unclear

*Data extraction: yes; study selection: unclear; **less than ten studies regarded diabetes mellitus

All risk of bias assessments were performed by two independent researches (JWa, HN). All results were displayed in a table and the results were colored differently, dependent on the positive, negative or any other non-significant influence of diabetes on the outcomes (survival, periimplantitis, osseointegration, augmentation). In addition, the studies in the table were colored differently if an influence of any supportive therapy, the glycemic control or the duration of diabetes mellitus has been reported.

## Results

### Study selection

One guideline from 2016 to the topic of dental implants and diabetes mellitus, in which the authors of this study (JWi, HN) play a key role, was identified.

A total of 177 potentially relevant titles and abstracts were found by the electronic search and additional evaluation of reference lists. During the first screening, 95 publications were excluded based on the title and keywords. In addition, 15 titles of clinical studies were excluded based on abstract evaluation. In total, 66 full-text articles were thoroughly evaluated, containing of clinical studies (*n* = 41) and reviews (*n* = 25). Ten titles had to be excluded at this stage, because they did not fulfil the inclusion criteria of the present systematic review.

56 articles (40 clinical studies and 16 reviews and meta-analyses) went into qualitative assessment by tabulating the study characteristics, implant related parameters and diabetes related parameters. Ten studies had to be excluded although they matched the inclusion criteria. One study had to be excluded, because diabetes was not used as possible variable for implantation related complications [65]. Nine studies of aggregated literature had to be excluded, because they were narrative without systematic literature research ([66–74] Fig. 1). No meta-analysis was performed, due to limited number of studies, heterogenic study design and incompletely reported data, such as type of diabetes therapy, quality of glycemic control and duration of disease. The quantitative data synthesis could not be performed in the way necessary for meta-analysis.

Regarding the clinical studies, the majority (*n* = 20) of the 40 studies were retrospective, eight had a cross-sectional study design. Eleven were prospective and one study was a randomized controlled trial. The main characteristics of the included studies are given in Table 6.

**Table 6:** List of the included clinical studies and its main characteristics

Study (author/year)	Study type	No. of patients	Age (mean)	Time of examination	Duration of study [months]	Number of implants	Survival rate [%]	Diabetestype	Control	Diabetes therapy	Glycemic control [HbA1c %]	Duration of diabetes mellitus
Eskow et al. (2017) [10]	Retrospective	24	59.7 ± 9.6	k.A.	34	59	98.6 (1 year); 96.6 (2 years)	2	No controlgroup	n.d.	“Poorly controlled”; 9.55 ± 1.0%	14.2 ± 7.7 years
Ormianer et al. (2018) [11]	Retrospective	169	55.9 ± 10.474	1995–2015	104	1112	94	2	n.d.	n.d.	“Moderately controlled” < 8%; “well-controlled” up to 7%	At least 2 years
Castellanos-Cosano et al. (2019) [12]	Retrospective	346	56.12 ± 12.15	2014–2017	48	44,415	n.d.	n.d.	No controlgroup	n.d.	n.d.	n.d.
Alrabiah et al. (2019) [13]	Cross-sectional	79	Prediabetic group: 54.3 ± 3.6; nondiabetic group: 51.2 ± 2.4	n.d.	60	80	100	Prediabetes	Nondiabetic group	n.d.	Prediabetic group: 6.1[5.9–6.3]%; nondiabetic group: 4.1[4–4.8]%	Prediabetes diagnosis 5.4 ± 0.2 years
Sghaireen et al. (2020) [14]	Retrospective	257	Diabetic group: 62.41 ± 13.62y; nondiabetic group: 59.24 ± 29.36 y	2013–2016	36	742	Diabetic group: 90.18; nondiabetic group: 90.95	n.d.	HbA1c < 6.5%	n.d.	“Well controlled” 6.5–8%; no further information + L8	n.d.
Papantonopoulos et al. (2017) [15]	Cross-sectional	72	61.9 ± 11.1	2014–2015	n.d.	237	n.d.	n.d.	Nondiabetic Clusters	n.d.	n.d.	n.d.
Atarchi et al. (2020) [16]	Cross-sectional	1343	61.66 ± 12.77	2002–2017	n.d.	2323	n.d.	n.d.	Nondiabetic group	n.d.	< 8%;“controlled diabetics”	n.d.
Alasqah et al. (2018) [17]	Cross-sectional	86	Diabetic group: 57.6 ± 5.5; nondiabetic group: 61.6 ± 4.3 y	n.d.	72	172	n.d.	2	Nondiabetic group; HbA1c 5.3 ± 0.3%	n.d.	“Well controlled” diabetes group: 4.8 ± 0.2%; nondiabetic group: 5.3 ± 0.3%	10.1 ± 3.5 years
Singh et al. (2020) [18]	Retrospective	826	n.d.	n.d.	120	1420	n.d.	n.d.	n.d.	n.d.	n.d.	n.d.
Al Zahrani et al. (2019) [19]	Prospective	67	Well controlled diabetics: 54.6 ± 9.9; poorly controlled diabetics: 53.8 ± 7.9	2009–2011	84	124	99 (7 years)	2	Poorlycontrolled diabetics	“Well controlled” diabetics: controlled by diet and anti-diabetic drugs (w or w/o insulin); “poorly controlled” diabetics: no control by diet or drugs	“Well controlled” diabetics: ≤ 6.0% “poorly controlled” diabetics: > 8.0%	Well controlled: 6.6 years; poorly controlled: 11.8 years
Erdogan et al. (2015) [20]	Prospective	24	Diabetic group: 52.5 ± 7.3; nondiabetic group: 49.5 ± 9.3	k.A.	12 (and more)	43	100	2	n.d.	All diabetic patients on active treatment (oral therapy, insuline, combination)	6.7 ± 0.3%	8.2 ± 3.5 years
Oztel et al. (2017) [21]	Retrospective	177	60.2 ± 15.1	2011–2013	36	302	95	n.d.	n.d.	n.d.	n.d.	n.d.
Gomez-Moreno et al. (2015) [22]	Prospective	67	Groups: HbA1c ≤ 6%: 60 ± 7.2; HbA1c = 6.1–8%: 59 ± 8.1; HbA1c = 8.1–10%: 62 ± 6.8; HbA1c ≥ 10.1%:64 ± 5.6	n.d.	36	67	n.d.	2	Four groups: HbA1c ≤ 6%; HbA1c = 6.1–8%; HbA1c = 8.1–10%; HbA1c ≥ 10.1%	n.d.	Four groups: HbA1c ≤ 6%; HbA1c = 6.1–8%; HbA1c = 8.1–10%; HbA1c ≥ 10.1%	n.d.
Dogan et al. (2015) [23]	Prospective	20	Diabetic group: 53.54 ± 4.01; nondiabetic group: 52.14 ± 3.93	2010–2011	7	39	n.d.	2	HbA1c 4.87 ± 0.53%	All diabetic patients: oral antidiabetics, exclusion criteria: insulin-therapy	“Well controlled”: 6.37 ± 1.28%	“At least 5 years”
Okamoto et al. (2018) [24]	Retrospective	289	Complications group: 62.8 ± 2.6; no complications group: 54.7 ± 13.1	2006–2013	0.75	298	100	n.d.	n.d.	n.d.	n.d.	n.d.
Al Amri et al. (2015) [25]	Prospective	91	HbA1c ≤ 6%: 48.5(45–52); HbA1c = 6.1–8%: 50.1(46–55); HbA1c = 8.1–10%: 50.5(45–59);	k.A.	24	n.d.	n.d.	2	HbA1c < 6%	k.A.	Three groups: HbA1c ≤ 6% (controls included); HbA1c = 6.1–8%; HbA1c = 8.1–10%	n.d.
de Araujo Nobre et al. (2016)	Retrospective	70	59(41–80)	1999–2007	60	352	89.8; group with type 1 diabetes mellitus: 80; group with type 1 diabetes mellitus: 90.5	1 and 2	No controlgroup	“Treated”; no further information	n.d.	n.d.
Al Amri et al. (2017) [26]	Retrospective	108	Immediately loaded group: 50.6 ± 2.2; conventional loading group 51.8 ± 1.7	n.d.	24	108	100	2	No controlgroup	n.d.	No initial HbA1c; At 12- and 24-month follow-up, the mean HbA1c levels in group 1 (immediately loading) and 2 (conventional loading) were 5.4%(4.8–5.5%) and 5.1%(4.7–5.4%) and 5.1%(4.7–5.2%) and 4.9%(4.5–5.2%)	Immediately loaded group: 9.2 ± 2.4 years; conventional loaded 8.5 ± 0.4 years
Al Amri et al. (2017) [27]	Prospective	45	Diabetic group: 42.4(40–46); nondiabetic group: 41.8(39–44)	n.d.	24	45	n.d.	2	HbA1c < 4.5% (visually, boxplot)	Antihyperglycemic drugs, dietary control	“Well controlled”: no exact data given, but visually (boxplots) the baseline HbA1c is significantly higher in the diabetic group (visually < 7%) than in nondiabetic control group (visually < 4.5%)	14.5 ± 0.7 months
Soh et al. (2020) [28]	Retrospective	89	n.d.	2019–2020	3	n.d.	n.d.	n.d.	n.d.	n.d.	n.d.	n.d.
Mohanty et al. (2018) [29]	Cross-sectional	208	n.d.	n.d.	96–120	425	Diabetic group: 70.7, periodontitis group: 83.3, smokers group: 80.9, bruxism group:86.3	n.d.	n.d.	n.d.	n.d.	n.d.
Aguilar-Salvatierra et al. (2016) [30]	Prospective	85	Group 1: 57 ± 3.8; group 2: 57 ± 3.8; group 3: 61 ± 1.9	n.d.	48	85	Group 1: 100; group 2: n.d.; group 3: 86.3	2	Three groups: HbA1c ≤ 6%; HbA1c = 6.1–8%; HbA1c = 8.1–10%	Oral hypoglycemic agents with similar doses	Three groups: HbA1c ≤ 6%; HbA1c = 6.1–8%; HbA1c = 8.1–10%	n.d.
Rekawek et al. (2021) [31]	Retrospective	286	n.d.	2006–2012	60 (and more)	748	n.d.	n.d.	HbA1c < 8%	k.A.	> 8% “uncontrolled diabetes”	n.d.
Jagadeesh et al. (2020) [32]	Retrospective	342	n.d.	n.d.	24	580	87.5	n.d.	n.d.	n.d.	n.d.	n.d.
Kandasamy et al. (2018) [33]	Retrospective	200	47.5	n.d.	96–180	650	n.d.	n.d.	n.d.	n.d.	n.d.	n.d.
Pedro et al. (2017) [34]	Prospective	23	71.05(65–80)	2009–2013	48	57	n.d.	n.d.	n.d.	n.d.	n.d.	n.d.
Yadav et al. (2018) [35]	RCT	88	Flap group: 54.35 ± 14.51; Flapless group: 57.82 ± 13.99	2013–2015	30	88	n.d.	2	n.d.	“Controlled” (under medication)	Inclusion criterion: HbA1c less than 7%; Flap group: 6.59 ± 0.42%; Flapless group: 6.75 ± 0.30%	Flap group: 6.55 ± 4.01 years; Flapless group: 6.91 ± 3.86 years
Khan et al. (2016) [36]	Retrospective	83	n.d.	2010–2015	n.d.	220	86,8	n.d.	n.d.	n.d.	n.d.	n.d.
French et al. (2021) [37]	Retrospective	4247	53.8 ± 13.5	1995–2019	54	10,871	98.9 (3 years), 98.5 (5 years), 96.8 (10 years), and 94.0 (15 years)	Mellitus	n.d.	n.d.	n.d.	n.d.
Alberti et al. (2020) [38]	Retrospective	204	57.3 ± 13.7	2005–2018	68	929	Diabetic group: 96.51, nondiabetic group: 94.74	1 and 2	n.d.	Diet:5, Metformin:7, Insuline:3, Sulfonylureas:1, Metformin + Sulfonylureas:3, Metformine + Pioglitazone + Glicazide:1	6.40 ± 0.36% (isurgery)	n.d.
Krebs et al. (2019) [39]	Retrospective	106	70.9(45–91)	1991–1997	227	274	n.d.	Mellitus	n.d.	n.d.	n.d.	n.d.
Dalago et al. (2017) [40]	Retrospective	183	59.3	1998–2012	68	938	98.3	n.d.	n.d.	n.d.	n.d.	n.d.
De Araújo Nobre et al. (2017) [41]	Prospective	22,009	48.5 ± 15.6	2012–2015	24	n.d.	n.d.	n.d.	n.d.	n.d.	n.d.	n.d.
Mayta-Tovalino et al. 2019) [42]	Retrospective	431	n.d.	2006–2017	132	1279	82.02	n.d.	n.d.	n.d.	n.d.	n.d.
Kissa et al. (2020) [43]	Cross-sectional	145	58.3	04/2017–12/2017	77	642	n.d.	Mellitus	n.d.	n.d.	n.d.	n.d.
Krennmair et al. (2018) [44]	Prospective	85	56.7 ± 11.2	2007–2009	60	295	99	2	n.d.	n.d.	≤ 7,5% controlled (> 7.5% = Exclusion criteria)	n.d.
Al-Sowygh et al. (2018) [45]	Cross-sectional	93	Three diabetic groups; group 1: 51.5(46–57), group 2: 53.7 (42–56), group 3: 55.9 (49–59); nondiabetic group: 50.1(41–53)	n.d.	n.d.	148	n.d.	2	Non-diabetic individuals with HbA1c < 6% (mean:5.8%)	n.d.	Group 1: HbA1c 6.1–8%(mean:6.7%); group 2: HbA1c 8.1–10%(mean:9.2); group 3: HbA1c > 10%(mean:11.4);	Group 1: 10.7(7–11.2) years, group 2: 9.4(8–10.6) years, group 3: 12.6(9.9–14.1) years
Corbella et al. (2020) [46]	Retrospective	112	57.3 ± 13.7	2004–2019	52	344	91.69 (12 years)	Mellitus	No controlgroup	n.d.	n.d.	n.d.
Al Amri et al. (2017) [47]	Prospective	24	Prediabetic group: 44.5(41–49); nondiabetic group: 43.3(39–47)	n.d.	12	24	100	Prediabetes	HbA1c: (baseline:) 4.4 ± 0.2%	n.d.	Baseline: Prediabetic group:6.1 ± 0.4; nondiabetic group:4.4 ± 0.2; follow-up values given	n.d.
Weinstein et al. (2020) [48]	Cross-sectional	248	63.4 (women); 62.5 (men)	Unclear	5	1162	n.d.	n.d.	n.d.	n.d.	n.d.	n.d.

*n.d.* no data provided

### Diabetes and osseointegration

Osseointegration is the process of osseous healing and bone remodeling building an actual interface between the living bone tissue and the implant surface, after implant insertion. This process is crucial for implant stability as well as inflammation-free survival [8].

In a prospective clinical study, 22 implants were placed in diabetics and 21 implants in a healthy control group (12 patients each). The stability values were comparable both at the time of implant insertion (ISQ 55.4 ± 6.5 vs. 59.6 ± 4.1, *p* = 0.087) and when the implant was exposed after 4 months (ISQ 73.7 ± 3.5 vs. 75.7 ± 3.2, *p* = 0.148) [20]. In another retrospective case–control study, 257 subjects were included, 121 with and 136 without diabetes; diabetes was defined as well controlled with an HbA1c below 8%. Implant failure in the osseointegration phase was observed in 17 cases in the diabetes group (4.5%) and 16 cases in the control group (4.4%), so that a non-significant difference has been concluded (*p* = 0.365) [14].

High primary stability, sufficient osseointegration and healthy surrounding tissue are prerequisites for concepts such as immediate or early restoration of the implants with prosthetic restorations. Immediate loading in patients with type 2 diabetes was investigated in two studies. In the retrospective cohort study with 108 diabetics, the immediately loaded implants showed an identical survival as those after 3 months with delayed loading (100% each) [66]. Next, in a prospective clinical study, the diabetic patients were divided into two groups based on the HbA1c value (Hba1c 6.1–8% and 8.1–10%) and compared with a control group with an HbA1c ≤ 6%. The implant survival rate in the control group and the group with an HbA1c between 6.1 and 8% was 100%, the group with an HbA1c of 8.1–10% showed an implant survival rate of 95.4% [30].

Regarding the question of osseointegration in prediabetes, one study could show similar success rates of implant healing in prediabetes as in the healthy collective [47].

### Diabetes and peri-implantitis

As diabetes mellitus is today seen as a systemic parainflammatic status [75] that is known to be associated with periodontitis and tooth loss [76], it is clear that the question of an increased risk of developing peri-implantitis in these patients is the subject of current research.

Thus, 23 studies could be included which contain a statement on peri-implantitis and diabetes mellitus or prediabetes. In fact, the conclusions on the influence of hyperglycemia on peri-implant inflammation are still heterogeneous. 12 clinical studies (1× cross-sectional study, 5× prospective, 6× retrospective) showed no increased risk of developing peri-implantitis with manifest diabetes mellitus [17, 23, 24, 27, 34, 38–41, 44, 46, 77]. On the other hand, six studies indicated an increased risk of peri-implant inflammation, with the highest determined relative risk being given as 8.65 [15, 28, 31, 48]. Two of these publications showed this especially in poorly controlled diabetes mellitus with an HbA1c > 8% with increased probing depths, bleeding on probing and peri-implant bone resorption [19, 45]. In five studies, no clear conclusion could be drawn from the data obtained, so that the question of an increased risk was not answered [10, 25, 33, 43, 64]. However, the available aggregated literature consistently concluded that diabetes mellitus represents a risk factor for the development of peri-implant inflammation, although most studies point to a lack of high-quality and long-term studies on this research area [8, 50, 51, 54–56, 58, 60–63].

Furthermore, two studies examined the effect of regular professional oral hygiene measures on the incidence of peri-implant inflammation in diabetics. In addition to a reduction in the clinical indicators of peri-implantitis, both studies could also show an improvement in the HbA1c value in the longitudinal course [25, 48].

Besides, two studies were included on the question of the influence of prediabetes on peri-implantitis. The prospective study by Al-Amri et al. with 24 test persons (12 prediabetic metabolic condition, 12 healthy) showed comparable clinical and radiological peri-implant findings in a 1-year observation interval, so that no increased risk was concluded [26]. The cross-sectional study by Alrabiah et al. with 79 subjects, however, indicated a higher incidence of peri-implant inflammation (probing depths, bleeding on probing, plaque index and peri-implant bone resorption) in prediabetes [13].

### Diabetes and implant survival

The results regarding diabetes and implant survival are heterogeneous. Five studies showed no negative influence [10, 11, 38, 42, 44], two showed a non-significant [29, 36] and six a significantly negative influence of diabetes on implant survival [12, 16, 32, 37]. For example, the study of Alberti et al. [38] showed no significant difference of the implant survival after 10 years in patients with diabetes (survival rate of 96.5%) compared to patients without diabetes mellitus (survival rate of 94.8%), whereas the study of French et al. [37] identified diabetes mellitus with a hazard ratio of 2.25 as a risk factor for implant failure in a multivariate analysis, implicating an over two times higher risk for failure of dental implants in patients with diabetes mellitus. In addition, eight aggregated literature references could be included on this question, whereby in seven publications, it was concluded that diabetes mellitus does not seem to have a significant influence on implant survival [8, 51, 52, 55–58, 63]. This includes two meta-analyses. The first meta-analysis demonstrated a relative risk of implant loss in these patients of 1.43, indicating a 43% higher risk for implant loss in patients with diabetes. Even though this corresponds with a confidence interval of 0.54–3.82 and a *p* value of *p* = 0.07, to a statistically insignificant increase in risk [51]. The other meta-analysis calculated a similar relative risk of 1.39 with a confidence interval of 0.58–3.30, which is also not statistically significant with a *p* value of *p* = 0.46 [58].

Two further studies were included that examined implant survival in prediabetes. Both, the cross-sectional and prospective studies, showed a similar level of implant loss in the prediabetic and the control group [13, 47].

### Diabetes and bone augmentation

We could identify one prospective study, that evaluated the effect of diabetes mellitus on maxillary sinus augmentation. Krennmair et al. performed a sinus lift with two-stage implant placement in a prospective study with a 5-year observation interval. In the evaluation, diabetics with an HbA1c < 7.5% were included and compared with non-diabetics. There was no difference in terms of bone augmentation, implant survival or peri-implant bone alteration [44]. A study on prediabetes and bone augmentation was not identified.

### Influence of quality of glycemic control

Two studies were included that demonstrated an influence of the quality of the blood sugar control on therapy with dental implants. In the cross-sectional study by Al-Sowygh et al. 93 patients were divided into four groups based on the HbA1c (< 6%, 6.1–8%, 8.1–10%, > 10%). It was found that with increasing HbA1c a significant deterioration in the clinical indicators for peri-implantitis could be observed. A significant difference could be shown in the group comparison of diabetic patients with a HbA1c 6.1–8% and > 8.1% [45]. The work by Eskow et al. comes to a comparable conclusion. They could show a positive correlation between the HbA1c value and peri-implant mucositis and implant loss [10]. Likewise, three meta-analyses were included in the aggregated literature. One analysis could show a positive correlation of the HbA1c and the bleeding on probing, but not with probing depths [50]. The other two analyses, on the other hand, showed no association between increased HbA1c and implant loss [57] or a correlation of HbA1c with clinical parameters of peri-implant complications [58].

### Influence of duration of diabetes disease

Information on the duration of the disease were given in 10 of 40 studies. The information remained descriptive in all studies. Therefore, no correlation of the duration of the disease and the possible influence on the implant therapy could be found.

### Influence of supportive therapy

The use of perioperative antibiotic prophylaxis and disinfecting mouthwash was reported in almost every study. No publication focused on the effect of an adjuvant anti-infective therapy on implant success in prediabetic or diabetic patients.

## Conclusions

This update was carried out on the basis of the publication of a large number of new studies in recent years, regarding dental implant insertion and possible complications in patients with diabetes mellitus in the last years. Therefore, for this update we could include a total number of 56 titles, consisting of 40 clinical studies and 16 titles of aggregated literature. This high number is an indication of the actuality and high interest in this research area and the large number of scientific questions that remain unanswered. Despite the large number of scientific publications, the level of evidence is not always high and the results are sometimes very heterogeneous. Furthermore, although the review process is quality assessed and indepentently performed by two of the reviewers (JWa, HN), but still is no automated, fully objective process.

In Germany around 7 billion people suffer from diabetes mellitus, with an estimated number of at least 2 billion cases on top [78]. In addition, prediabetes represents an increasing health problem with an annual conversation rate of 5–10% in manifest type 2 diabetes mellitus [79] and as it could be shown in follow-up data, the risk of developing diabetic microvascular complications is not only increased in patients with type 2 diabetes mellitus but already in patients with prediabetes [80].

Accordingly, diabetes mellitus should be recognized as a potential risk factor for delayed osseointegration, the occurrence of peri-implant inflammation and poor implant survival and has to be taken into account in patient management and treatment decisions as well as follow-up care.

Previous studies clearly showed, that poorly controlled HbA1c can have negative effects on osseointegration and primary stability of dental implants, as we could already show in our review in 2016 [8], but the information on osseointegration in well controlled diabetes mellitus is still heterogeneous. Nevertheless, the indication for immediate and early loading should be viewed critically, especially in poorly controlled diabetes mellitus.

The influence of diabetes mellitus on the development of peri-implant inflammation in the early phase is unclear due to the heterogeneous data situation. In contrast, the risk seems to increase over time after implantation. Hence, risk-adapted follow-up care should be carried out after implant placement.

There are no significant differences in the survival rates in the first few years of diabetics compared to the healthy comparison group. However, in the long term, the risk of implant loss seems to be increased as previous studies could show [81–83]. Referring to prediabetes, this seems to have no influence on dental implant loss at all.

Furthermore, the evidence available on the influence of the quality of blood glucose control on the success of implant therapy is heterogeneous and there is insufficient evidence on the possible influence of the duration of the illness of diabetes mellitus on implant therapy. The final assessment regarding the influence of the duration of diabetes mellitus is also still pending.

In conclusion the results of our systematic review and the included literature more or less confirmed earlier knowledge in this field [8]. It has to be mentioned, that especially the preoperative preparation and evaluation of possible risk factors as well as the postoperative visits and recall gains importance, as the implant insertion itself is already highly standardized and perioperative anti-infective procedures are carried out in most cases. In addition, we included literature regarding oral rehabilitation with dental implants in prediabetic conditions in this review. Whereas, prediabetes seems to have no influence on implant survival rates at all.

Taking the existing evidence together, it can be concluded that oral rehabilitation with dental implants in patients with prediabetes and diabetes mellitus is a safe and predictable procedure. In times of precision medicine, a precise indication and a risk-adapted approach and adopted recall system for patients with prediabetes and type 2 diabetes mellitus is inevitable and provides a high probability for implant success.

## Data Availability

All data generated or analysed during this study are included in this published article.
